# On the quantum circuit implementation of modus ponens

**DOI:** 10.1038/s41598-024-65224-9

**Published:** 2024-06-20

**Authors:** Songsong Dai

**Affiliations:** School of Electronics and Information Engineering, Taizhou University, Taizhou, 318000 Zhejiang China

**Keywords:** Quantum information, Mathematics and computing

## Abstract

The process of inference reflects the structure of propositions with assigned truth values, either true or false. Modus ponens is a fundamental form of inference that involves affirming the antecedent to affirm the consequent. Inspired by the quantum computer, the superposition of true and false is used for the parallel processing. In this work, we propose a quantum version of modus ponens. Additionally, we introduce two generations of quantum modus ponens: quantum modus ponens inference chain and multidimensional quantum modus ponens. Finally, a simple implementation of quantum modus ponens on the OriginQ quantum computing cloud platform is demonstrated.

## Introduction

**Figure 1:**
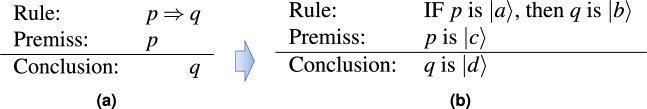
From modus ponens to quantum modus ponens. (**a**) Form of modus ponens. (**b**) The architectire exhibition of quantum modus ponens. The rule consists of an antecedent “*p* is ${|{a}\rangle }$” and a consequent “*q* is ${|{b}\rangle }$”.

The term “quantum” is spreading continuously in interdisciplinary scientific literature. Studying the quantum counterparts of existing classical models is an inspiration for technological advancement^1–8^. Inference is omnipresent, appearing in physics, mathematics, and daily life. The basic rule of classical inference model is the modus ponens (see Fig. 1a): if *p* is true and *p* implies *q*, then *q* is true. For example, if *p* represents “The cat is alive” and *q* represents “The mouse is dead,” then *p* is true and the implication $p\Rightarrow q$ holds, it follows that “The mouse is dead”.

When it comes to quantum world, however, Schrödinger’s cat is simultaneously alive and dead. Then, is the mouse alive or dead? Typically, we know that the rule remains unchanged, then, in some sense, we may infer that “the mouse is also simultaneously alive and dead” from the new premiss. However, this inference is visibly somewhat arbitrary and lacks justification. In this work, we introduce the quantum modus ponens (QMP) which comes from the consideration of the quantum counterpart of modus ponens in classical logics. As sketched in Fig. 1b, our QMP inference form has a quantum premiss and a quantum rule which includes a quantum rule antecedent and a rule consequent. The word “quantum” in the QMP refers to the quantization of true values of propositions. In classical MP, every proposition is assigned a definite truth value, either ’true’ or ’false’. Truth-value assignment in QMP is assigned a qubit. The superposition state $a_{0}{|{0}\rangle }+a_{1}{|{1}\rangle }$ represents a proposition that involves of a certain degree of uncertainly. According to quantum probability, $|a_{1}|^2$ stands for the probability of the proposition being false, while $|a_{0}|^2$ represents the probability of the proposition being true. This is denoted as $\texttt {P}({|{\psi }\rangle })=|a_{1}|^2$ for any ${|{\psi }\rangle }=a_{0}{|{0}\rangle }+a_{1}{|{1}\rangle }$.

## Results

### Principle and circuit of QMP

In Classical MP, $q=p\wedge (p\rightarrow q)$ which states that if *p* is true and *p* implies *q*, then *q* is true. We extended this to QMP using quantum probability, which asserts that the probability of the conclusion being true is equal to the probability of the premiss being true multiplied by the probability of the rule being true.

First, consider the rule “IF *p* is ${|{a}\rangle }$, then *q* is ${|{b}\rangle }$”. What is the probability of this rule being true? In classical logic, $p\rightarrow q=1$ contains three cases: $p=q=1$, $p=q=0$, and $p=0$ and $q=1$. In this sense, the probability of the rule being true calculated as follows,

1 $$\begin{aligned} \texttt {P}({|{a\rightarrow b}\rangle })=\texttt {P}({|{a}\rangle })\texttt {P}({|{b}\rangle })+(1-\texttt {P}({|{a}\rangle }))(1-\texttt {P}({|{b}\rangle }))+ (1-\texttt {P}({|{a}\rangle }))\texttt {P}({|{b}\rangle })=1-\texttt {P}({|{a}\rangle })+\texttt {P}({|{a}\rangle })\texttt {P}({|{b}\rangle }). \end{aligned}$$

**Figure 2:**
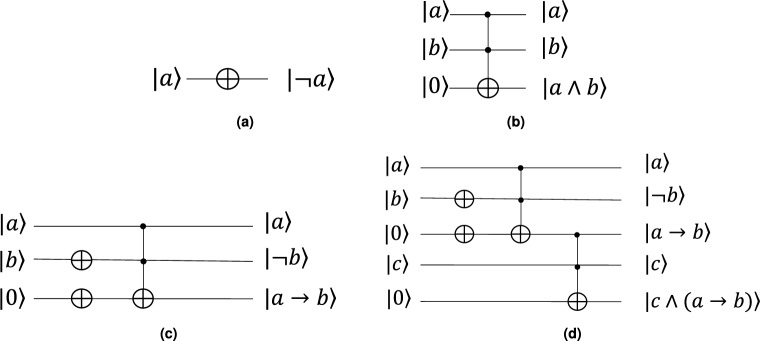
Quantum circuits of rule and quantum modus ponens. The circuit implementation is based on the NOT (**a**) and AND (**b**) gates. First, we calculate the true value of the rule (**c**) and then determine the inference’s conclusion (**d**).

We introduce a quantum circuit that utilizes the NOT and AND gates to derive the rule’s result (see Fig. 2a and b), with the AND gate being obtained through the Toffoli gate. The quantum cost of Fig. 2c is 5 since the quantum cost of a Toffoli gate is 5^9^.

We then consider the conclusion derived from this rule and the premise “*p* is ${|{c}\rangle }$”, which is true only if both the rule and the premise are true. In this sense, the probability of the conclusion being true calculated as follows,

2 $$\begin{aligned} \texttt {P}({|{d}\rangle })=\texttt {P}({|{c}\rangle }) \texttt {P}({|{a}\rangle }\rightarrow {|{b}\rangle })=\texttt {P}({|{c}\rangle })-\texttt {P}({|{a}\rangle })\texttt {P}({|{c}\rangle })+\texttt {P}({|{a}\rangle })\texttt {P}({|{b}\rangle })\texttt {P}({|{c}\rangle }). \end{aligned}$$

Clearly, we have the following property

3 $$\begin{aligned} \texttt {P}({|{d}\rangle })\le \texttt {P}({|{c}\rangle }). \end{aligned}$$

This property implies that the probability of the conclusion being true cannot be greater than the probability of the premise being true.

Now, we present a quantum circuit to implement QMP (see Fig. 2d) with 2 NOT gates and 2 Toffoli gate. The quantum cost of Fig. 2d is 10. It can also be used to simulate classical modus ponens inference.

### Generalization to a chain of inferences

The quantum analogy of the chain of inferences is an intriguing extension of MP inference. It involves a sequence of logical deductions: if $p_{1}$ is true and $p_{1}$ implies $p_{2}$, up to the point where $p_{n-1}$ implies $p_{n}$, then $p_{n}$ is also true. This can be formally expressed as

4 $$\begin{aligned} p_{n}=p_{1}\wedge (p_{1}\rightarrow p_{2})\wedge \cdots \wedge (p_{n-1}\rightarrow p_{n}). \end{aligned}$$

**Figure 3:**
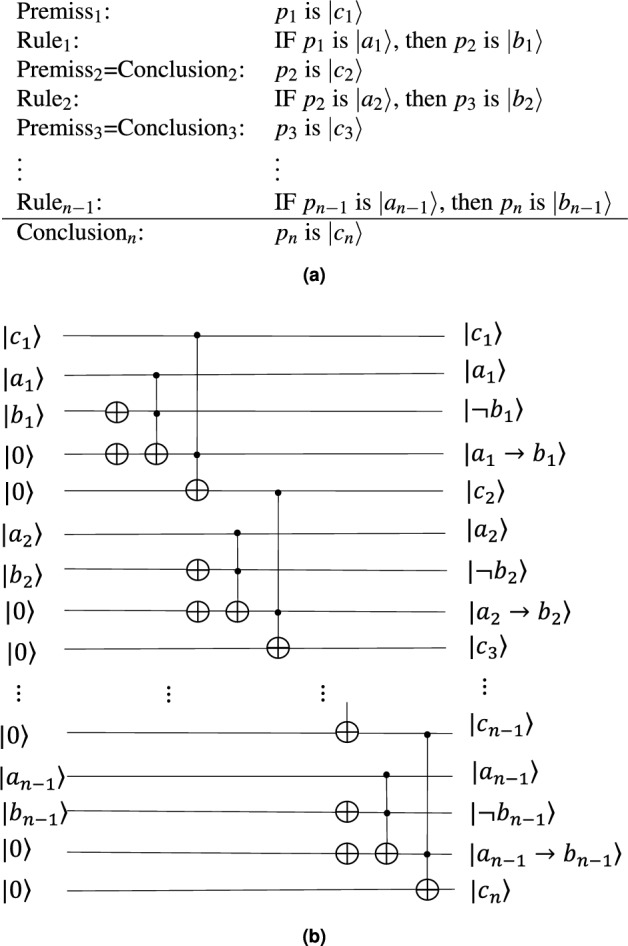
Form and circuit of a chain of inference. (**a**) A chain includes one premise and $n-1$ rules. Each premise$_i$ and rule$_i$ deduce a conclusion$_{i+1}$, which then becomes the premise$_{i+1}$ for the next inference. (**b**) The circuit diagram shows how to implement the inference chain using $4n-3$ qubits, $2n-2$ NOT gates, and $2n-2$ Toffoli gates.

We will now delve into the quantum counterpart of this chain of inferences (see Fig. 3a).

The conclusion “$p_{n}$ is ${|{c_{n}}\rangle }$” is derived from the initial premise “$p_{1}$ is ${|{c_{1}}\rangle }$” and $n-1$ rules $p_{i}\rightarrow p_{i+1}$ ($i=1,...,n-1$), which holds true only when all rules and the initial premise are true. Therefore, the probability of the conclusion being true can be calculated as follows:

5 $$\begin{aligned} \begin{aligned} \texttt {P}({|{c_{n}}\rangle })&=\texttt {P}({|{c_{1}}\rangle }) \texttt {P}({|{a_{1}}\rangle }\rightarrow {|{b_{1}}\rangle })\cdots \texttt {P}({|{a_{n-1}}\rangle }\rightarrow {|{b_{n-1}}\rangle })\\&=\texttt {P}({|{c}\rangle }) (1-\texttt {P}({|{a_{1}}\rangle })+\texttt {P}({|{a_{1}}\rangle })\texttt {P}({|{b_{1}}\rangle }))\cdots (1-\texttt {P}({|{a_{n-1}}\rangle })+\texttt {P}({|{a_{n-1}}\rangle })\texttt {P}({|{b_{n-1}}\rangle })). \end{aligned} \end{aligned}$$

Clearly, we have the following property

6 $$\begin{aligned} \texttt {P}({|{c_{n}}\rangle })\le \texttt {P}({|{c_{n-1}}\rangle })\le \cdots \le \texttt {P}({|{c_{1}}\rangle }). \end{aligned}$$

This property implies that as more rules are applied, the likelihood of the conclusion being true decreases.

Now we present a quantum circuit to implement the chain of QMP inferences (see Fig. 3b) with $2n-2$ NOT gates, and $2n-2$ Toffoli gates. The quantum cost of Fig. 3b is 10n-10. It provides all the intermediate conclusions and the truth values for each rule.

### Generalization to *n* premises, one conclusion

Another fascinating generalization of modus ponens is multidimensional modus ponens. This concept posits that if propositions $p_{1},\cdots ,p_{n}$ are true and it holds that $p_{1},\cdots ,p_{n}$ imply *q*, then *q* is true. Formally, this generalization can be expressed as

7 $$\begin{aligned} q=p_{1}\wedge \cdots \wedge p_{n}\wedge (p_{1}\wedge \cdots \wedge p_{n}\rightarrow q). \end{aligned}$$

**Figure 4:**
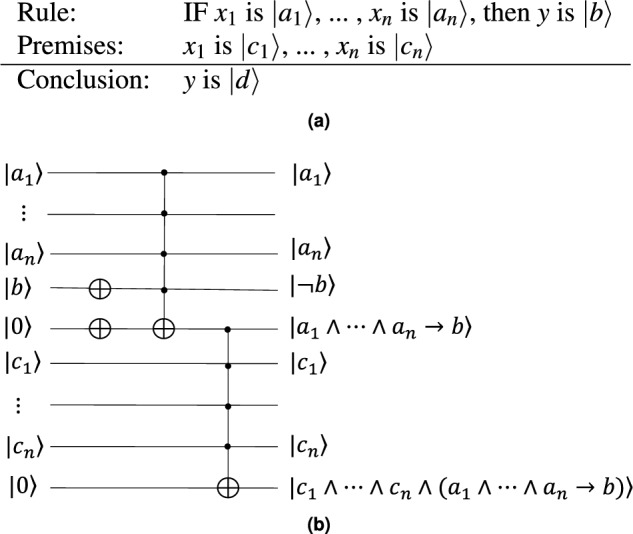
General form and circuit of multidimensional quantum modus ponens. (**a**) One conclusion can be inferred from *n* premises and one rule that includes *n* antecedents and one consequent. (**b**) The circuit diagram shows how to implement the inference chain using $4n-3$ qubits, $2n-2$ NOT gates, and $2n-2$ Toffoli gates. The circuit diagram shows how to implement the multidimensional quantum modus ponens using $2n+3$ qubits, two NOT gates, and two $(n+2)$-qubit Toffoli gates.

Delving into the quantum counterpart of this notion is our next focus, and you can find a visual representation in Fig. 4a.

The conclusion “*q* is ${|{d}\rangle }$” is derived from the *n* premises “$x_{1}$ is ${|{c_{1}}\rangle }$, ... , $x_{n}$ is ${|{c_{n}}\rangle }$” and one rule with *n* antecedents and one consequent “IF $x_{1}$ is ${|{a_{1}}\rangle }$, ... , $x_{n}$ is ${|{a_{n}}\rangle }$, then *y* is ${|{b}\rangle }$”. The conclusion is true only when all premises and the rule are true. Therefore, the probability of the conclusion being true can be calculated as follows:

8 $$\begin{aligned} \begin{aligned} \texttt {P}({|{c_{n}}\rangle })&=\texttt {P}({|{c_{1}}\rangle })\cdots \texttt {P}({|{c_{n}}\rangle }) \texttt {P}({|{a_{1}}\rangle }\wedge \cdots \wedge {|{a_{n-1}}\rangle }\rightarrow {|{b}\rangle })\\&=\texttt {P}({|{c_{1}}\rangle })\cdots \texttt {P}({|{c_{n}}\rangle }) (1-\texttt {P}({|{a_{1}}\rangle })\cdots \texttt {P}({|{a_{n}}\rangle })+\texttt {P}({|{a_{1}}\rangle })\cdots \texttt {P}({|{a_{n}}\rangle })\texttt {P}({|{b_{1}}\rangle })). \end{aligned} \end{aligned}$$

Clearly, we have the following property

9 $$\begin{aligned} \texttt {P}({|{d}\rangle })\le \texttt {P}({|{c_{1}}\rangle })\cdots \texttt {P}({|{c_{n}}\rangle }). \end{aligned}$$

As a corollary, we have $\texttt {P}({|{d}\rangle })\le \texttt {P}({|{c_{i}}\rangle })$ for each $i=1,...,n$. We see that the more premises we use, the less likely the conclusion is to be true.

Now we present a quantum circuit to implement the multidimensional modus ponens (see Fig. 4b) with two NOT gates, and two $(n+2)$-qubit Toffoli gates. The quantum cost of a $(n+1)$-qubit Toffoli gate is 32n-120^10^, then quantum cost of Fig. 3b is 64n-176.

**Figure 5:**
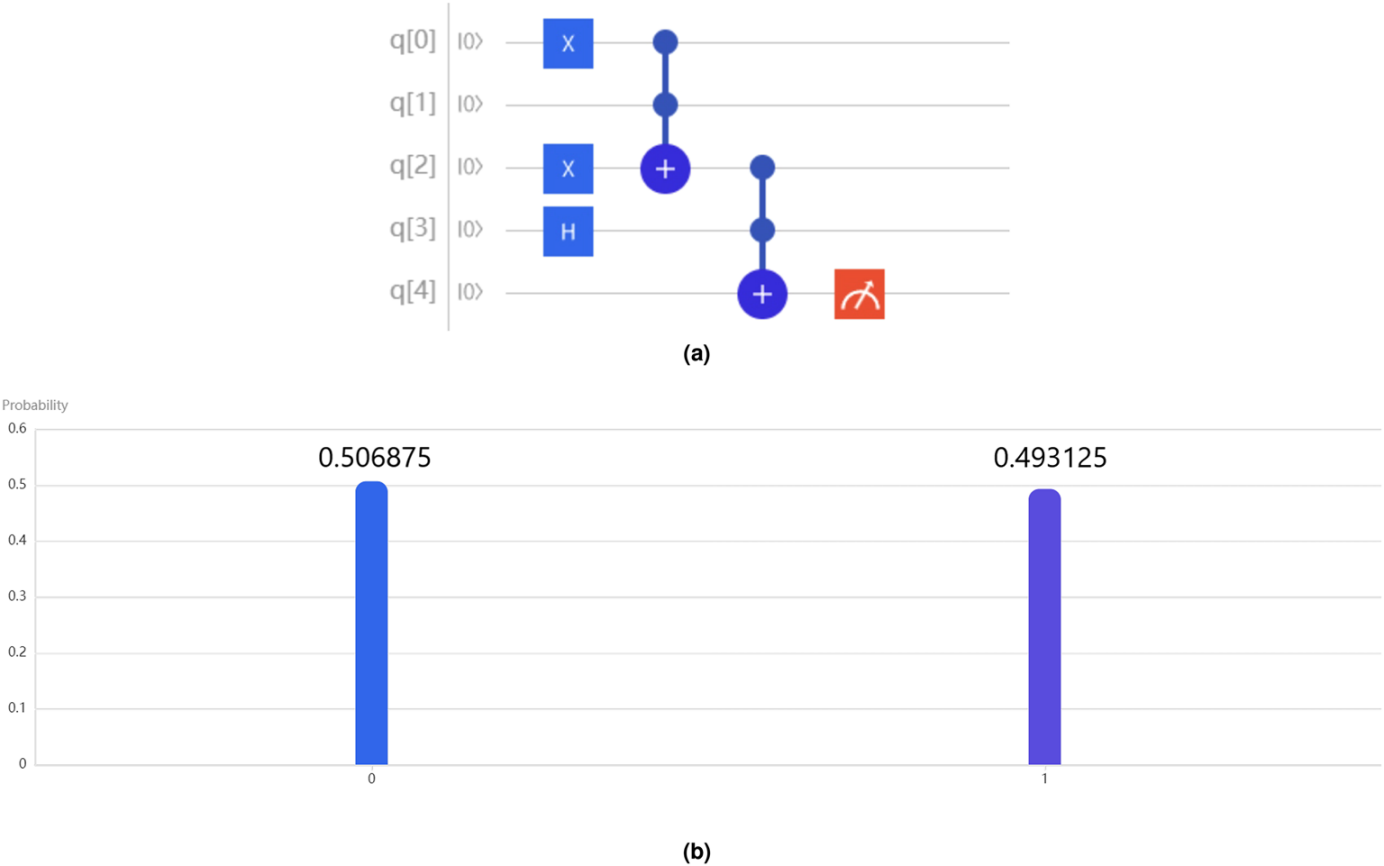
An example of quantum modus ponens. (**a**) Our circuit is about the quantum modus ponens with the rule “IF *p* is ${|{1}\rangle }$, then *q* is ${|{0}\rangle }$” and the premiss “ *p* is $\frac{{|{0}\rangle }+{|{1}\rangle }}{\sqrt{2}}$”. (**b**) The output is [0.506875, 0.493125], indicating that the probability of *q* being false is 0.506875, while the probability of *q* being true is 0.493125.

### A sample quantum computer implementation

Let us consider the example in the Introduction section, the rule is “If the cat is alive, then the mouse is dead,” and the premise is that “The cat is simultaneously alive and dead.” So what is the conclusion? To represent the truth of the rule’s antecedent, we denote ${|{1}\rangle }$. Similarly, we use ${|{1}\rangle }$ to signify the truth of the rule’s consequent. Furthermore, the premise being simultaneously true and false is symbolized by $\frac{{|{0}\rangle }+{|{1}\rangle }}{\sqrt{2}}$.

This implementation is executed using the OriginQ quantum computing cloud platform^11^. Additionally, the quantum circuit for this example is depicted in Fig. 5a. The initial state is ${|{0}\rangle }{|{0}\rangle }{|{0}\rangle }{|{0}\rangle }{|{0}\rangle }$. Firstly, by using NOT gate on *q*[0] and *q*[2], and Hadamard gate on *q*[3], then the initial state is converted to ${|{1}\rangle }{|{0}\rangle }{|{1}\rangle }\frac{{|{0}\rangle }+{|{1}\rangle }}{\sqrt{2}}{|{0}\rangle }$. Secondly, a Toffili gate is used on *q*[0], *q*[1] and *q*[2] and the quantum state is still ${|{1}\rangle }{|{0}\rangle }{|{1}\rangle }\frac{{|{0}\rangle }+{|{1}\rangle }}{\sqrt{2}}{|{0}\rangle }$. Here *q*[0] and *q*[1] represent the states of antecedent and consequent of the rule, respectively. And then the output of *q*[2] is result of the rule. Thirdly, a Toffili gate is used on *q*[2], *q*[3] and *q*[4], and the quantum state becomes ${|{1}\rangle }{|{0}\rangle }{|{1}\rangle }\frac{{|{0}\rangle }+{|{1}\rangle }}{\sqrt{2}}\frac{{|{0}\rangle }+{|{1}\rangle }}{\sqrt{2}}$. Here the input of *q*[3] is state of the premiss and the output of *q*[4] is state of the conclusion. In a perfect scenario, the conclusion “the mouse is dead” is $\frac{{|{0}\rangle }+{|{1}\rangle }}{\sqrt{2}}$, i.e., simultaneously true and false. The process further involves measuring the 5th qubit of the output. The result of executing the program of Fig. 5a on OriginQ quantum platform is shown in Fig. 5b. As it can be seen, the probability of the state being ${|{0}\rangle }$ after the measurement is 0.506875, while the probability of the state being ${|{1}\rangle }$ is 0.493125, note that the number of repeated tests is 8000.

## Discussion

As mentioned by Feynman^2^, quantum mechanical systems are better simulated by quantum computers than classical computers. In this perspective, reasoning with quantum data can be achieved using quantum inference, which involves collections of rules capable of mapping quantum inputs to quantum outputs. The goal achieved by this research is the proof of the quantum computers feasibility in performing inference involving quantum premises and quantum conclusions. This research initiates the extension of MP inference to the quantum scenario, and formalizes the MP inference chain and multidimensional MP inference accordingly, providing quantum circuits as well. There is still much research to be done, particularly in the applications of IF-Then rules. For example, utilizing a large set of IF-Then rules to build a quantum inference system and applying it to pattern recognition and decision making.

## Data Availability

All data generated or analysed during this study are included in this published article.
